# The Potential Role of Heparin in Patients With COVID-19: Beyond the Anticoagulant Effect. A Review

**DOI:** 10.3389/fphar.2020.01307

**Published:** 2020-08-21

**Authors:** Lucia Gozzo, Pierluigi Viale, Laura Longo, Daniela Cristina Vitale, Filippo Drago

**Affiliations:** ^1^ Clinical Pharmacology Unit/Regional Pharmacovigilance Centre, University Hospital of Catania, Catania, Italy; ^2^ Infectious Diseases Unit, Department of Medical and Surgical Sciences, Policlinico Sant’Orsola, University of Bologna, Bologna, Italy; ^3^ Department of Biomedical and Biotechnological Sciences, University of Catania, Catania, Italy

**Keywords:** COVID-19, coagulopathy, heparin, pleiotropic activity, clinical trials

## Abstract

Severe acute respiratory syndrome coronavirus 2 (SARS-CoV-2) infection is responsible of variable clinical manifestations, ranging from no symptoms to severe pneumonia with acute respiratory distress syndrome, septic shock, and multi-organ failure resulting in death. To date no specific antiviral drug have been approved for COVID-19, so the treatment of the disease is mainly focused on symptomatic treatment and supportive care. Moreover, there are no treatments of proven efficacy to reduce the progression of the disease from mild/moderate to severe/critical. An activation of the coagulation cascade leading to severe hypercoagulability has been detected in these patients, therefore early anticoagulation may reduce coagulopathy, microthrombus formation, and the risk of organ damages. The role of heparin in COVID-19 is supported by a lot of studies describing its pleiotropic activity but it must be proven in clinical trials. Several protocols have been designed to assess the risk-benefit profile of heparin (low-molecular-weight or unfractionated heparin) in hospitalized subjects. Although prophylactic doses may be adequate in most patients, it is important to wait the results of clinical trials in order to define the appropriate effective dose able to improve disease outcome.

## Introduction

The clinical manifestations of the severe acute respiratory syndrome coronavirus 2 (SARS-CoV-2) infection range from asymptomatic infection to severe pneumonia with acute respiratory distress syndrome (ARDS), septic shock, and multi-organ failure resulting in death (Wang Y. et al., 2020).

A large Chinese epidemiological study showed that among 44,672 confirmed cases, 80.9% were mild, 13.8% severe, and 4.7% critical. The fatality rate for critical patients was 49%, higher in patients with comorbidities (cardiovascular disease 10.5%, diabetes 7.3%, chronic respiratory disease 6.5%, hypertension 6.0%, cancers 5.6%) than those without comorbidities (0.9%) (Wang Y. et al., 2020). Laboratory findings of Corona Virus Disease 19 (COVID-19) include lymphopenia with depletion of CD4 and CD8 lymphocytes, prolonged prothrombin time, elevated lactate dehydrogenase (LDH), D-Dimer, alanine transaminase, C-reactive protein (CRP), and creatinine kinase (Huang et al., 2020; Wang D. et al., 2020).

One of the most important mechanisms underlying the deterioration of disease is the cytokine storm (Shimabukuro-Vornhagen et al., 2018). This clinically severe phase is accompanied by high level of pro-inflammatory molecules, such as interferons α and β, and IL-6 (Mehta et al., 2020).

Severe disease is also complicated with coagulopathy and disseminated intravascular coagulation (DIC) has been reported in the majority of deaths (Tang et al., 2020a). Patients with progressive, severe COVID-19 infection with acute lung injury or ARDS have very high D-dimer and fibrinogen levels, related to a hypercoagulable state. Moreover, severe and critically ill COVID patients with prolonged immobilization are inherently at high risk of venous thromboembolism (VTE) and some patients who require mechanical ventilation may have acute pulmonary embolism (PE) or deep vein thrombosis (DVT), even without strong predisposing risk factors.

Thus, an early anticoagulation, which blocks uncontrolled blood clotting and reduce micro-thrombus formation, would lower the risk of major organ disfunction. Accordingly, even if the risk-benefit ratio has not been established, the World Health Organization (WHO) recommended in these patients thrombo-prophylaxis with either unfractionated or low molecular weight heparin (LMWH) (Driggin et al., 2020; WHO, 2020b; WHO, 2020a).

Aim of this work is to describe the link between inflammation, immune activation, and coagulopathy and the hypothetical pleiotropic role of heparin in COVID-19.

## Inflammation, Sepsis, and Coagulopathy

A variety of disorders (sepsis, systemic inflammatory conditions, trauma, malignant disease) lead to activation of the coagulation system, up to the most extreme form of DIC, and microvascular thrombosis is a frequent complication of critical illness conditions (Dhainaut et al., 2005; Ito, 2014).

Inflammation and coagulation are clearly linked by different molecular signals and their interactions play a major role in the pathophysiology of sepsis and DIC (Levi and Poll, 2015; Li and Ma, 2017).

Acute infections, including viral ones, induce a systemic inflammatory response and coagulation disruption (Subramaniam and Scharrer, 2018). The process is complex and multifactorial, involving cellular disruption and plasmatic elements of the hemostatic system and of the innate immune system to the pathogen (Gando et al., 2016). Thrombosis under certain circumstances plays a major physiological role in immune defense. The coagulation system and innate immunity (the so-called *immunothrombosis system*) play a beneficial role in early host defense against pathogens (Delvaeye and Conway, 2009; Fiusa et al., 2015), limiting microbial dissemination, protecting blood vessels, promoting recruitment and activation of leukocytes through fibrin, fibrinogen, and their degradation products, and stimulating cellular immune responses at the infection sites. Moreover, intravascular thrombi produce a distinct compartment where antimicrobial peptides can be concentrated and kept in contact with pathogens. However, aberrant or uncontrolled *immunothrombosis* may be harmful, determining an imbalance between pro-coagulant and anticoagulant mechanisms (Ito, 2014).

Multiple pathogenetic mechanisms have been identified in the coagulation cascade activation, and involving endothelial cells, von Willebrand factor, Toll-like receptor, and tissue-factor pathway (van Gorp et al., 1999; Ito, 2014). The effect is the deregulated thrombin generation, further worsened by the impairment of anticoagulant and fibrinolytic systems.

The pro-inflammatory mediators activate coagulation, which in turn promotes inflammatory activity (Opal, 2000; Russell, 2006; Hunt, 2014). In particular, inflammation promotes coagulation by leading to intravascular tissue factor expression, inducing the expression of leukocyte adhesion molecules on the endothelial cell, and down-regulating the fibrinolytic pathways by the up-regulation of plasminogen activator inhibitor-1 (PAI-1). On the other hand, thrombin stimulates inflammatory response in a self-propagating feedback loop.

The simultaneous impairment of pro-coagulant pathways and fibrinolytic systems as a result of systemic inflammation lead to platelet activation and fibrin deposition (Simmons and Pittet, 2015; Levi and van der Poll, 2017). It has been demonstrated that the most important mediators for orchestrating this imbalance during sepsis are cytokines (Levi et al., 1997), such as interleukin-1 (IL-1), IL-6, and tumor necrosis factor-α (TNF-α), but also denatured DNA and cationic proteins, such as histones, released from damaged cells (McDonald et al., 2017)[21].

The final result of the uncontrolled activation of the coagulation system is multiple organ dysfunction (Iba and Levy, 2018; Li X. et al., 2020).

Moreover, it is relevant in the pathogenesis of specific organ damage, such as ARDS (MacLaren and Stringer, 2007; Frantzeskaki et al., 2017). The lung coagulopathy is related to a localized tissue factor-mediated thrombin generation, and depression of bronchoalveolar plasminogen activator-mediated fibrinolysis, mediated by the PAI-1 increase (Glas et al., 2013; Ozolina et al., 2016).

Thus, the involvement of the hemostatic system in severe COVID-19 is not surprising, being well documented that inflammation and sepsis are initiators of DIC (Voves et al., 2006). The most typical findings in patients with COVID-19 and coagulopathy are an increased D-dimer level, a modest decrease in platelet count, and a prolongation of the prothrombin time (Levi et al., 2020). The pattern is therefore different to that typically seen in sepsis, in which thrombocytopenia is more severe, and D-dimer not very high (Levi and Scully, 2018). In particular, markedly elevated D-dimer has been detected and associated with higher intensive care unit (ICU) admission and mortality, likely reflecting coagulation activation, cytokine storm development, and organ failure (Guan et al., 2020; Huang et al., 2020; Tang et al., 2020b; Zhou et al., 2020). Furthermore, post-mortem examinations show vascular thrombosis in small vessels of the lungs (Carsana et al., 2020; Menter et al., 2020; Wichmann et al., 2020), suggesting that the COVID-19 coagulopathy can include, besides a low-grade of DIC, a so-called “Pulmonary Intravascular Coagulopathy-PIC” (Belen-Apak and Sarialioglu, 2020; Fogarty et al., 2020; McGonagle et al., 2020), a localized pulmonary thrombotic micro-angiopathy determining organ damage (Levi et al., 2020).

It is believed that the coagulation cascade in COVID-2019 can be activated through the well-known mechanisms reported above, which lead to the deregulated thrombin generation both systemically and locally in the lungs, resulting in the deposition of fibrin with subsequent tissue damage and micro-angiopathy (Li T. et al., 2020). Moreover, SARS-CoV-2 would directly damage vascular endothelial cells through angiotensin-converting enzyme 2 (ACE2), which could represent the first injury triggering the abnormal coagulation in particular in the lung (Li H. et al., 2020). However, other studies showed that ACE2 pulmonary expression is restricted to type II pneumocytes, and is nearly absent in endothelial (McGonagle et al., 2020; Rivellese and Prediletto, 2020). In this context, the strict contact between type II pneumocytes and the pulmonary vascular network, and the severe local inflammatory reaction, is likely to drive the generalized pulmonary hypercoagulable state seen in patients with COVID-19 (Li H. et al., 2020; McGonagle et al., 2020; Rivellese and Prediletto, 2020). Nevertheless, the mechanisms contributing to coagulopathy in COVID-19 have to be comprehensively clarified yet.

## Treatment Strategies

To date, treatment of coagulopathy/DIC has been focused on the target of the primary associated pathology (Levi and Scully, 2018). This is limited in the case of COVID-19, due to the lack of approved antiviral drug treatment, so the management of patients is mainly focused on symptomatic and supportive care. Moreover, there are no treatments of proven efficacy to reduce the progression of the disease from mild/moderate to severe/critical, in particular counteracting the cytokine storm (Chen et al., 2020). However, reducing the release or activity of pro-inflammatory mediators can prevent or reverse the uncontrolled hyper-inflammation, thereby improving the condition of patients and a lot of drugs with this aim are under evaluation in clinical trials.

The use of anticoagulants for patients with severe COVID-19 has been recommended by expert consensus and by WHO (Driggin et al., 2020; WHO, 2020b).

The International Society of Thrombosis and Haemostasis (ISTH) introduced a new category identifying an earlier phase of sepsis-associated DIC, called “sepsis‐induced coagulopathy” (SIC) (Iba et al., 2019). In this case or in patients with markedly elevated D-dimers, LMWH at prophylactic dose should be considered (Tang et al., 2020a).

The optimal thrombo-prophylactic regimen in patients with COVID-19 is unknown (Driggin et al., 2020). Given drug-drug interaction with direct oral anticoagulants and some anti-viral regimens, heparins, either unfractionated or low molecular weight, may be preferred.

Accurate patient assessment is necessary to balance the individual risk of thrombosis and bleeding. Therapeutic anticoagulation is not required unless another indication for therapeutic anticoagulation is documented (e.g. VTE, atrial fibrillation, or mechanical valve). Moreover, evidence of coagulopathy/DIC and especially elevated D-dimer levels observed even in early phase of PIC might be useful to guide therapeutic decision (Lillicrap, 2020).

Prophylactic dose LMWH is recommended for all hospitalized COVID-19 patients in the absence of contraindications.

However, standard prophylactic regimens may be insufficient in severe and critically ill patients with variable thromboembolic/bleeding risk, and monitoring of anti-Xa activity may be considered when LMWH is used in these patients (Duranteau et al., 2018).

In cases where there are no contraindications, empiric therapeutic anticoagulation has been proposed by the American Society of Hematology in the following cases (Ash, 2020):

- intubated patients who develop sudden clinical and laboratory findings highly consistent with PE;
- patients with physical findings consistent with thrombosis (superficial thrombophlebitis, peripheral ischemia or cyanosis, thrombosis of dialysis filters, tubing, or catheters);
- patients with respiratory failure, particularly when D-dimer and/or fibrinogen levels are very high, in whom PE or microvascular thrombosis is highly suspected and other causes are not identified (e.g., ARDS, fluid overload).

A normal level D-dimer level provides reasonable confidence that anticoagulation should continue at prophylactic doses.

However, the efficacy and safety of anticoagulation as well as the appropriate dose regimen able to improve disease outcome in patients with COVID-19 have yet to be defined in clinical trials.

## Pharmacological Properties of Heparin and Clinical Evidence in COVID-19

Although primarily employed for its anticoagulant properties, it is known that heparin possesses anti-inflammatory, immunomodulatory, anti-viral, and anti-complement activity which may offer benefit beyond the anti-coagulation (Davidson et al., 2002; Hoppensteadt et al., 2008; Young, 2008; Ludwig, 2009; Li et al., 2012; Li et al., 2014; Li et al., 2015; Li and Ma, 2017; Thachil, 2020).

Heparin is a member of a family of polyanionic polysaccharides called glycosaminoglycans (Young, 2008). It remains one of the most important anticoagulant drugs in clinical practice, currently used for the prevention and treatment of venous thrombosis and PE, the management of arterial thrombosis in patients with acute myocardial infarction and in the prevention of re-thrombosis after thrombolysis, and the prevention of thrombosis in extracorporeal circuits and hemodialysis.

The mechanisms behind its pleiotropic effect are complex and not completely understood.

Its polyanionic nature allows to bind sites proteins such as antithrombin III, but also cytokines, chemokines, growth factors, adhesion molecules, cytotoxic peptides, tissue destructive enzymes, involved in inflammation (Day et al., 2004). Thus, the binding of acute phase and complement proteins may contribute to the anti-inflammatory activity of heparin (Weiler et al., 1992; Young et al., 1997).

Indeed, even if the binding of released cytokines may protect them from proteolytic degradation, heparin may alter the secondary and tertiary structure of cytokines and prevent the binding to their specific receptors (Balasubramanian and Ramanathan, 2000; Mummery and Rider, 2000; Jayanthi et al., 2017), thus, influencing their biological activity, limiting accumulation of inflammatory cells and activation and subsequent tissue damage. When given in pharmacological doses, exogenous heparin and heparinoids demonstrated to attenuate tissue damage, neutralizing a variety of mediators released from inflammatory cells (Elsayed and Becker, 2003).

In line with this assumption, a large number of studies have revealed that LMWH reduce the release and the biological activity of IL-6 and IL-8 (Qian et al., 2014; Shastri et al., 2015; Li et al., 2016; Liu et al., 2019).

In addition, heparin binding to P-selectin showed to inhibit leukocyte adhesion to endothelial cells, independently by its anticoagulant activity (Lever et al., 2000).

The dysfunction of endothelial cells and the reduction of glycocalyx are key characteristics of sepsis. Heparin, as a heparan sulphate (HS) analogue, may reconstitute the protective layer of proteoglycans to restore the natural vascular barrier (Nelson et al., 2008). The protective function on the endothelial tight junctions has been demonstrated in a model of lung damage induced by lipopolysaccharide, where heparin administration decreased edema and vascular leakage (Liu et al., 2019).

Moreover, the protective responses observed with heparin in experimental models of sepsis seem to be mediated by blocking the pro-inflammatory signaling pathways regulated by MAPK, NF-κB, and STAT3 (Iba and Levy, 2018; Li X. et al., 2020). It has been demonstrated that heparin is readily bound and internalized into the cytosolic compartment, where it can prevent the NF-κB translocation to the nucleus through the binding of the positively charged nuclear localization sequence (Letourneur et al., 1995; Akimoto et al., 1996; Dudas et al., 2000). Blocking of this transcriptional factor can reduce inflammatory gene activation and regulate the production of pro-inflammatory cytokines, chemokines, and adhesion molecules.

A novel immune-modulating mechanism of heparin related to blockage of circulating histones has been studied ***in vitro*** and in septic mouse models (Wildhagen et al., 2014). It is noteworthy that extracellular histones released from dead cells play important role in cellular damage and are robustly associated with endothelial dysfunction, organ dysfunction and even death during sepsis (Xu et al., 2009; Ekaney et al., 2014; Iba et al., 2015). Heparin demonstrated a strong affinity for extracellular histones and prevents their interaction with platelets, a potential mechanism contributing to the regulation of inflammation (Fuchs et al., 2011; Alhamdi et al., 2016).

Finally, the putative antiviral role of heparin has been studied in experimental models. Thanks to its polyanionic nature, heparin can bind to several proteins, such as cell surface glycoproteins and thus inhibit herpes simplex virus attachment (Shukla and Spear, 2001). Furthermore it has been demonstrated that in zika virus infection it prevents virus-induced cell death (Ghezzi et al., 2017).

Interestingly, ***in vitro*** and ***in vivo*** experimental studies have shown that human coronaviruses utilize heparin sulfate proteoglycans for attachment to target cells (Milewska et al., 2014), and interaction between the SARS-CoV-2 Spike S1 protein receptor binding domain (SARS-CoV-2 S1 RBD) and heparin has been recently showed, supporting the role of heparin in the therapeutic armamentarium against COVID-19 beyond the anticoagulant effect (Courtney Mycroft-West et al., 2020).

However, the exact benefit and safety of heparin as anti-inflammatory and antiviral agent in clinical setting are yet to be defined and conflicting results have been reported by previous clinical trials.

According to systematic reviews and meta-analyses regarding the use of heparin as a potential treatment for patients with sepsis, treatment with low doses of heparin is associated with significantly reduced 28-day mortality in sepsis (Liu et al., 2014; Wang et al., 2014; Zarychanski et al., 2015; Fan et al., 2016).

Another meta-analysis shows a reduction of the risk of 7-day and of 28-day mortality, and a significant improvement of PaO2/FiO2 ratio in patients with ARDS treated with high-dose LMWH (Li et al., 2018), demonstrating that treatment with heparin may be helpful in mitigating the pulmonary coagulopathy found in ARDS.

The existing evidence on the use of heparin to prevent or treat thrombotic complications in COVID-19 derives from retrospective and observational data.

Recently, a retrospective cohort study analyzed the relieving effect of LMWH in patients with COVID-19, to investigate the anti-inflammatory effects of heparin and the delay of disease progression (Shi C et al., 2020). Compared to the control group, patients treated with heparin had an improvement of hypercoagulability, a reduction of IL-6 and neutralization of its biological activity, and an increase in the percentage of lymphocytes. A large retrospective cohort showed lower mortality in COVID-19 patients treated with heparin, even after adjustment for age and gender (OR 95% CI 0.55, 0.37–0.82; p = 0.003), saturation of oxygen <90%, and temperature >37°C (OR 0.54, 0.36–0.82; p = 0.003), and use of concomitant medications (OR 0.42, 0.26–0.66; p < 0.001) (Ayerbe et al., 2020). Moreover, a recent observational study conducted in US found a reduced risk of mortality among patients (n = 786) hospitalized with COVID-19 who received anticoagulation (Paranjpe et al., 2020).

Randomized controlled trials are necessary to confirm these preliminary observations.

### Ongoing Clinical Trials in COVID-19

As reported on the COVID-19 clinical trials registry (http://www.covid-trials.org, 2020) which collects all trials from International Clinical Trials Registry Platform (Chinese Clinical Trial Registry, ClinicalTrials.gov, Clinical Research Information Service—Republic of Korea, EU Clinical Trials Register, ISRCTN, Iranian Registry of Clinical Trials, Japan Primary Registries Network, and German Clinical Trials Register), 16 clinical trials are ongoing (9/16 recruiting and 7/16 not-recruiting) to evaluate the effect of anticoagulation with heparin (low-molecular-weight—mainly enoxaparin—or unfractionated heparin) in hospitalized patients with COVID-19 (**Appendix 1**). More than 80% of these studies are open-label, randomized, two-arm trials, and at least 75% of protocols include a comparison between therapeutic anticoagulation (investigational arm) and thromboprophylaxis (control arm), in line with the uncertainty about the benefit/risk ratio of the two treatment strategies. As reported in **Appendix 2**, the primary outcome measures of heparin clinical trials are hard endpoints such as mortality or composite measure of clinical events and/or survival, as recommended by the WHO guidelines (WHO, 2020d).

Overall, almost 10,000 patients are expected to be enrolled. However, the completion of some studies (expected in the second half of 2020 and in 2021) would be difficult at least in European countries and China due to the reduction in the number of new cases and hospitalizations (WHO, 2020c).

## Conclusion

Coagulation activation has been reported in COVID-19, determining pathological changes speciﬁcally involving the lung microvasculature, and an increased risk of DVT, PE, and DIC in severe phase. The use of anticoagulants, in particular heparin, is recommended by expert consensus for patients with severe COVID-19, although a final guidance cannot be implemented yet.

There are several ways in which probably heparin administration can benefit patients with COVID-19, beyond the anticoagulant effect.

Although prophylactic doses may be adequate in most patients, it would be important to administer therapeutic dosage based on the individual risk of coagulopathy and thrombosis. To assess the efficacy and safety in patients with COVID-19 in clinical trials is crucial in order to find the appropriate effective dose of LMWH/UFH and improve disease outcomes. Different well-designed clinical trials (randomized, controlled, with appropriate outcome measures, even if not-blinded) are ongoing. However, the completion of trials and the consequent definition of risk/benefit profile of drugs candidate for COVID-19 would be complicated by the reduced (albeit strongly awaited) spread of the virus.

## Author Contributions

LG wrote the first draft of the manuscript. PV and FD checked and revised the draft manuscript. All authors contributed to the article and approved the submitted version.

## Conflict of Interest

The authors declare that the research was conducted in the absence of any commercial or financial relationships that could be construed as a potential conflict of interest.

## Appendix 1 Ongoing clinical trials with heparin in patients with COVID-19 (update May 28, 2020).

**Table:**

ID	Country	Treatment	Phase	Completion	Trial status	Design	Blinding	Arms	Patient setting	Size
2020-001709-21	France	Enoxaparin, tinzaparin, dalteparin, nadroparin	IV	NA	Recruiting	Randomized	Open-label	2	Hospital	550
2020-001823-15	France	Enoxaparin	IV	NA	Recruiting	Single-arm	Open-label	1	ICU	200
2020-001891-14	Spain	Enoxaparin	II	NA	Recruiting	Randomized	Open-label	2	Hospital	140
CHICTR2000030700	China	Enoxaparin	/	2020-Sep	Not recruiting	Randomized	Open-label	2	Hospital	60
CHICTR2000030701	China	Enoxaparin	/	2020-Sep	Not recruiting	Randomized	Open-label	2	Hospital	60
CHICTR2000030946	China	LMW heparin	IV	2020-Apr	Recruiting	Non-randomized	Unspecified	2	Hospital	120
NCT04344756	France	Tinzaparin, enoxaparin, dalteparin, unfractionated heparin	II	2020-Jul	Not recruiting	Randomized	Open-label	2	Hospital, ICU	808
NCT04345848	Switzerland	Enoxaparin Unfractionated heparin	III	2020-Nov	Recruiting	Randomized	Single	2	Hospital, ICU	200
NCT04354155*	United States	Enoxaparin	II	2022-Sept	Not recruiting	Single-arm	Open-label	1	Hospital	38
NCT04359277	United States	Enoxaparin Unfractionated heparin	III	2021-Apr	Recruiting	Randomized	Open-label	2	Hospital	1,000
NCT04360824	United States	Enoxaparin	IV	2021-Apr	Not recruiting	Randomized	Open-label	2	Hospital	170
NCT04362085	Canada	LMW heparin Unfractionated heparin	III	2020-Nov	Recruiting	Randomized	Open-label	2	Hospital	462
NCT04366960	Italy	Enoxaparin	III	2020-Aug	Recruiting	Randomized	Open-label	2	Hospital	2,712
NCT04367831	United States	Enoxaparin Unfractionated heparin	IV	2020-Nov	Recruiting	Randomized	Single	4	ICU	100
NCT04372589	Canada	Enoxaparin, tinzaparin, dalteparin, unfractionated heparin	/	2021-Jan	Not recruiting	Randomized	Open-label	2	Hospital	3,000
NCT04377997	United States	Enoxaparin Unfractionated heparin	II	2021-Jan	Not recruiting	Randomized	Open-label	2	Hospital, ICU	300

Four trials recently approved in Italy and not yet reported in the online registry are not included.

NA, not available; ICU, intensive care unit. *Pediatric subjects.

## Appendix 2 Main outcome measures of ongoing clinical trials with heparin in patients with COVID-19.

**Table:**

ID	Primary outcome measures
2020-001709-21	Onset of a symptomatic venous thromboembolic event, or symptomatic pulmonary embolism, or unexplained death when a pulmonary embolism cannot be excluded
2020-001823-15	Measurement of the anti-Xa activity of enoxaparin
2020-001891-14	Need for oxygen therapy escalation or invasive mechanical ventilation or mortality
CHICTR2000030700	Time to Virus Eradication
CHICTR2000030701	Time to Virus Eradication
CHICTR2000030946	Biochemical indicators
NCT04344756	Survival without ventilation (NIV or mechanical ventilation) in patients not requiring ICU who need for oxygen but no NIV or high flow. Ventilator free survival in patients with respiratory failure and requiring mechanical ventilation
NCT04345848	Composite outcome of arterial or venous thrombosis, disseminated intravascular coagulation, and all-cause mortality
NCT04354155	Safety of in-hospital thromboprophylaxis
NCT04359277	All-cause mortality, cardiac arrest, symptomatic deep venous thrombosis, pulmonary embolism, arterial thromboembolism, myocardial infarction, hemodynamic shock
NCT04360824	Mortality
NCT04362085	Composite outcome of ICU admission, non-invasive positive pressure ventilation, invasive mechanical ventilation, or all-cause death
NCT04366960	Incidence of venous thromboembolism
NCT04367831	Composite of being alive and without clinically-relevant venous or arterial thrombotic events at discharge from ICU
NCT04372589	Need for invasive mechanical ventilation or mechanical ventilation, and occurrence of death
NCT04377997	Composite efficacy endpoint of death, cardiac arrest, symptomatic deep venous thrombosis, pulmonary embolism, arterial thromboembolism, myocardial infarction, or hemodynamic shock

ICU, intensive care unit; NIV, non-invasive ventilation.
